# Prognostic impact of tumor-associated macrophages in pediatric Ewing sarcoma, National Cancer Institute (2010–2020)

**DOI:** 10.1186/s12957-026-04295-6

**Published:** 2026-03-27

**Authors:** Mustafa Mohamed Selim, Reem Ragab Hegazy, Mahitab Ibrahim Eltohamy, Nesreen Ali Ahmed

**Affiliations:** 1 Pediatric Oncology and Hematology Department, NCI, Cairo University, Kasr Al Eini Street, 4 Form El Khalig, Cairo, 11796 Egypt; 2 Department of Pathology, NCI, Cairo University, Cairo, Egypt; 3 Children’s Cancer Hospital, Cairo, CCHE-57357 Egypt

**Keywords:** ES, Tumor-associated macrophages, CD68, CD163 expression

## Abstract

**Background:**

Ewing sarcoma (ES) is the second most common primary malignant bone tumor in children and adolescents. Tumor-associated macrophages (TAMs) are a key component of the tumor microenvironment. While increased TAMs infiltration is linked to poor prognosis in various solid tumors, limited data are available regarding their role in sarcomas.

**Methods:**

This retrospective study aimed to evaluate the frequency, density, and prognostic impact of TAMs in pediatric patients with ES treated at the Pediatric Oncology Department, National Cancer Institute (NCI), Cairo University. Immunohistochemical analysis of CD68 and CD163 expression was performed on 74 tumor tissue samples from pediatric patients with ES, which helps us understand the density of TAMs.

**Results:**

The study cohort comprised 74 patients with a median age of 12 years (range: 0.5–18 years) and a male-to-female ratio of 0.8:1. The 5-year overall survival (OS) and event-free survival (EFS) rates were 48% and 42.8%, respectively. CD68 expression showed significant correlations with age at diagnosis (*p* = 0.035) and tumor size (*p* = 0.031) and near-significant associations with skeletal tumor location (*p* = 0.052) and complete response following induction chemotherapy (*p* = 0.054). CD163 expression was significantly associated with age at diagnosis (*p* = 0.046). However, neither CD68 (≤ 60 vs. > 60) nor CD163 (≤ 90 vs. > 90) levels showed significant correlation with survival outcomes (OS or EFS). Initial primary tumor site, tumor stage, post-induction chemotherapy disease status, and histological response to induction chemotherapy were significant predictor of survival outcome.

**Conclusions:**

TAM markers (CD68 and CD163) did not demonstrate a significant prognostic impact on survival. Larger-scale studies are warranted to more precisely determine the role of TAMs in pediatric ES.

## Background

ES is the second most common malignant bone tumor in children and adolescents, with a peak incidence between 10 and 19 years of age. Approximately 15% of cases occur in children under 10 years, with a slight male predominance [1, 2].

Despite advancements in multidisciplinary treatment—including surgery, radiotherapy, and systemic chemotherapy—the progression-free survival (PFS) rate for patients with localized ES remains limited to 60–70%. In contrast, patients presenting with metastatic disease at diagnosis continue to have a poor prognosis, with reported PFS rates ranging from only 18% to 30% [3].

Historically, prognostic factors have included clinical and pathological features such as the presence of metastases, response to chemotherapy, and tumor location, size, and volume. However, there is limited information on molecular and microenvironmental markers that could help guide risk assessment at the time of diagnosis. Recent evidence suggests that the tumor micro-environment (TME) plays an important role in cancer development and progression [4].

The TME comprises tumor cells, stromal components, vasculature, and immune cells. Among these, tumor-associated macrophages (TAMs), part of the innate immune system, have been shown to influence tumor progression and immune evasion in various sarcoma subtypes [5]. Macrophages can adopt either an M1-like (anti-tumorigenic) or M2-like (pro-tumorigenic) phenotype [6, 7]. In many solid tumors, a high density of M2-like TAMs is associated with poor prognosis [8].

Despite recent advances in immunotherapy, including immune checkpoint inhibitors such as anti-PD-1 agents, responses in sarcoma patients remain limited [9]. Consequently, alternative therapeutic strategies are being explored, including targeting components of the TME, particularly TAMs, which play a central role in cancer-related inflammation [6, 10].

This study was initiated to evaluate the density and frequency of TAMs and to assess their prognostic significance in pediatric patients with ES.

## Patients and methods

### Patients and study design

This retrospective cohort study included 74 pediatric patients with histologically confirmed ES, diagnosed and treated at the Pediatric Oncology Department, NCI, Cairo University, Egypt, between January 1, 2010, and December 31, 2020. Diagnosis was confirmed by a specialized pathologist at our institution.

Relevant clinical data were extracted from medical records, including patient demographics, clinical and pathological characteristics, laboratory and histopathological findings, treatment details (including systemic chemotherapy, surgical and radiotherapy modalities), treatment response, and outcome. Immunohistochemical evaluation for TAMs was performed using CD68 and CD163 markers.

### Inclusion and exclusion criteria

Eligible patients were under 18 years of age with de novo ES and available adequate tissue samples for analysis. Patients were excluded if they had received chemotherapy outside NCI or if their medical records were incomplete.

### Histopathological assessment

Formalin-fixed, paraffin-embedded tissue biopsies (*n* = 74) were retrieved from the archives of the Anatomic Pathology Department, NCI, Cairo University. Archived slides and diagnostic markers were re-evaluated to confirm diagnosis and ensure tissue adequacy for further immunostaining. Corresponding paraffin blocks were used to perform immunohistochemistry.

### Immunohistochemistry

Immunohistochemical staining was performed using monoclonal antibodies specific for TAM markers on pre-treatment specimens. CD68 expression was detected using a mouse anti-human monoclonal antibody (clone KP1; DAKO, Code M0814), while CD163 expression was evaluated using a mouse monoclonal antibody (clone MRQ-26; ROCHE). These antibodies were selected to identify and differentiate macrophage subpopulations within the tumor microenvironment.

### Analysis and scoring

All immunostained slides were evaluated blinded to information about the patients’ backgrounds or their prognoses. To avoid substantial bias in field selection, the representative fields of immunostaining were chosen at low magnification, and macrophage assessment followed a standardized sampling protocol. Multiple representative high-power fields, including hotspots and average-density regions, were analyzed to avoid contingency of field artifacts and ensure that macrophage density measurements reflected true spatial heterogeneity within the tumor microenvironment. The density of CD68- and CD163-positive cells was quantitatively assessed by light microscope CX31. Only viable tumor areas were considered, and areas of necrosis were neglected. Positively stained cells were counted in five randomly selected areas in a high-power field in the microscope. Positive reaction for CD68 was estimated by diffuse or granular cytoplasmic staining, while positive reaction for CD163 was estimated by cytoplasmic and membranous reaction. The macrophages were counted in an area of 1 mm², which was created from five fields at the magnification power of x40.

For scoring, 2 methods were adopted. The first method we followed [4] (Handl et al., 2018); the cases were then stratified into three levels according to the number of TAMs (the proportion of CD68 positive cells was scored as follows): (I) < 60 positive cells; (II) 61–130 positive cells; and (III) > 131 positive cells. Levels of CD163-positive cells were specified as (I) < 80 positive cells, (II) 81–140 positive cells, and (III) > 140 positive cells. The second method is by using the median for both CD68 and CD163.

The median-based method was defined as the primary analytical approach for CD68 and CD163, as it allowed an unbiased stratification of our cohort. Literature-based categorical cutoffs were additionally applied as exploratory analyses to facilitate comparison with prior reports and to test the robustness of our findings across different thresholds.

### Treatment protocol

Patients were treated according to the Children’s Oncology Group (COG) protocol AEWS0031 [11], which consists of alternating cycles of cyclophosphamide, doxorubicin, and vincristine (CDV) with ifosfamide and etoposide (IE). Chemotherapy was administered either every three weeks (non-interval compression) during 2010–2015 or every two weeks (interval compression) during 2016–2020. The CDV regimen included vincristine (2 mg/m², maximum dose 2 mg), doxorubicin (37.5 mg/m² on days 1–2), and cyclophosphamide (1.2 g/m²), alternating with IE consisting of ifosfamide (1.8 g/m²) and etoposide (100 mg/m² on days 1–5), for a total of 14 cycles. Filgrastim (5 µg/kg/day; maximum dose 300 µg) was administered following each cycle to support neutrophil recovery.

### Evaluation of treatment response and local control

Treatment response was evaluated based on the Response Evaluation Criteria in Solid Tumors (RECIST), version 1.1 as follows [12]: Complete Response (CR) was defined as the complete disappearance of all target lesions; Partial Response (PR) as a reduction in primary tumor volume by 50–99%; Stable Disease (SD) as less than a 50% reduction in measurable lesions with no new lesions; and Progressive Disease (PD) as the appearance of new lesions, a ≥ 25% increase in the size of measurable lesions, or conversion of previously negative bone marrow to positive.

Response was evaluated after induction chemotherapy (following 4 cycles in the standard schedule or 5 cycles in the interval-compressed schedule), after local control, and at the end of treatment. For analysis, patients were grouped into two response categories: objective response, which included CR and PR, and non-objective response, which included SD and PD. Patients with PD were considered for second-line chemotherapy to achieve disease control and facilitate surgical intervention.

Following induction chemotherapy, patients were assessed for the feasibility of surgical excision. If surgery was not feasible, or if only partial resection was possible, or if margins were positive, patients received local radiotherapy. Surgery was generally performed 2–3 weeks after the last chemotherapy cycle. Resection margin was categorized according to the Union for International Cancer Control; margin negative (R0), microscopically positive (R1), and grossly positive (R2) [13]. Surgery was omitted in patients with no residual disease or with unresectable residual tumors.

### Study endpoints

EFS was defined as the time from diagnosis to the occurrence of the first event, including disease progression, relapse, death, or last follow-up. OS was measured from the date of diagnosis to death from any cause or last follow-up. Refractory disease was defined as failure to achieve a response following initial therapy, while relapse was defined as a ≥ 25% increase in tumor size or the appearance of a new disease site after an initial response. The last date of follow-up for all patients was May 4, 2022.

### Statistical analysis

Data were managed and analyzed using the Statistical Package for the Social Sciences (SPSS), version 25. Continuous variables were tested for normality and presented as means with standard deviations (SD) or as medians with ranges, as appropriate. Categorical variables were summarized as frequencies and percentages. Comparisons between continuous variables were conducted using the Student’s t-test for normally distributed data and the Mann–Whitney U test for non-normally distributed data. Categorical variables were compared using the chi-square test or Fisher’s exact test, as appropriate. Survival outcomes were analyzed using the Kaplan–Meier method, and differences between survival curves were assessed with the log-rank test. Variables that were statistically significant in univariate survival analysis were entered into a multivariate Cox proportional hazard regression model using the forward likelihood ratio method. Hazard ratios (HRs) and its 95% confidence intervals (CIs) were used for risk estimation. All statistical tests were two-sided, and a *p*-value < 0.05 was considered statistically significant.

## Results

### Patient characteristics

A total of 74 patients were identified with adequate tissue blocks for immunohistochemical analysis and complete clinical records. The median age of the cohort was 12 years (range, 0.5–18 years), with a male-to-female ratio of 0.8:1. Among these patients, 59 (79.7%) had primary bone origin, while the remaining 15 (20.3%) had soft tissue origin. Within the primary bone-origin group, tumors arising in the extremities were more common than those located in the axial skeleton (44 patients, 74.6%, vs. 15 patients, 25.4%, respectively). The proportion of patients with smaller tumors (< 8 cm) was comparable to those with larger tumors (≥ 8 cm). Metastatic disease at diagnosis was present in 24 patients (32.4%). Among these, 14 patients (58.3%) had single-site metastasis (10 to the lungs, 2 to the bone marrow, and 2 to the bone), while 10 patients (41.7%) presented with multiple-site metastases (Table 1).

**Table 1:** Clinicopathological characteristics of studied patients

Characteristics (*n*, 74)	Number (%)
Age	
< 15 years	47 (63.5)
≥ 15 years	27 (36.5)
Gender	
Male	32 (43.2)
Female	42 56.8%)
Primary tumor site	
Extremity bone	44 (74.6)
Axial bone	15 (25.4)
Soft tissue	15 (20.3)
Tumor size	
< 8 cm	35 (50)
≥ 8 cm	35 (50)
Missing	4 (5.4)
Metastatic status	
Non metastatic	50 (67.6)
Metastatic	24 (32.4)
Number of metastatic sites (*n* = 24)	
Single site	14 (58.3)
Multiple sites	10 (41.7)
Chemotherapy given	
Every 2 weeks	50 (71.4)
Every 3 weeks	20 (28.6)
Disease status after induction treatment	
Objective response (13 CR − 29 PR)	42 (71.2)
Non-objective response (9 SD − 8 PD)	17 (28.8)
Not assessed	15
Local control type	
Surgery (3 upfront surgery − 21 delayed surgery)	24 (37.5)
Radiotherapy	24 (37.5)
Combined	16 (11.5)
Not done	10 (13.5)

Data presented as numbers and percentages as appropriate

*CR* complete response, *PR* partial response, *SD* stable disease, *PD* progressive disease

### Histopathological assessment of the cases

**Fig. 1:**
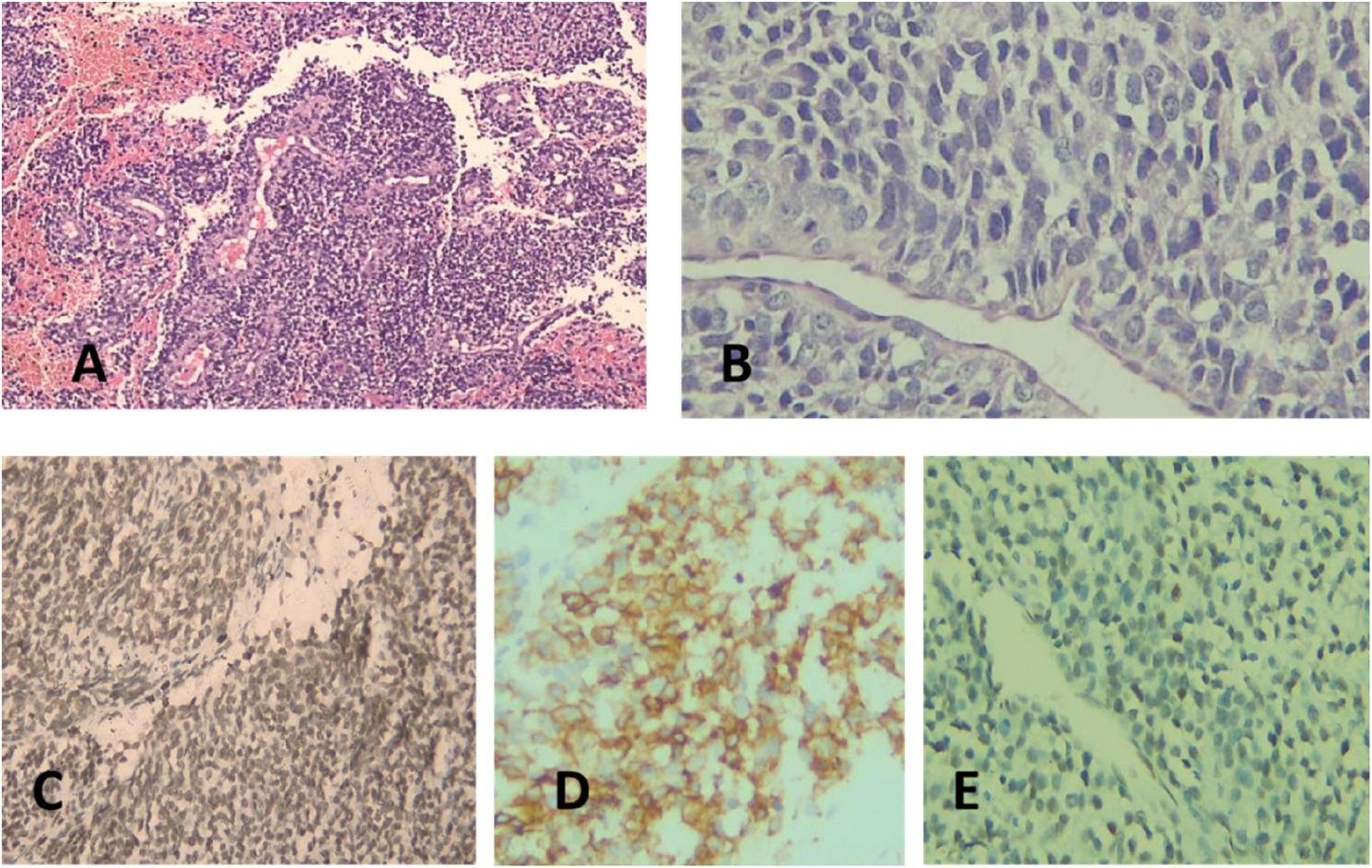
Histopathological assessment of the cases. **A**- Hematoxylin and Eosin-stained slide showing a case of Ewing sarcoma magnification (x200); **B**- Higher magnification showing sheets of small to intermediate round cells with vesicular nuclei with noticed anaplasia (x400); **C**- Immunohistochemical stained slide showing positive nuclear reaction to NK-X2 (x200); **D**- Immunohistochemical stained slide showing positive membranous reaction to CD99 (X400); **E**- Immunohistochemical stained slide showing positive nuclear reaction to FLI-1 (x200)

Archived biopsy slides from paraffin-embedded tissue at the time of diagnosis showed positive staining for the previously performed immunohistochemical markers CD99 and NKX2.2 (Fig. 1).

### CD68 and CD163 expression

**Fig. 2:**
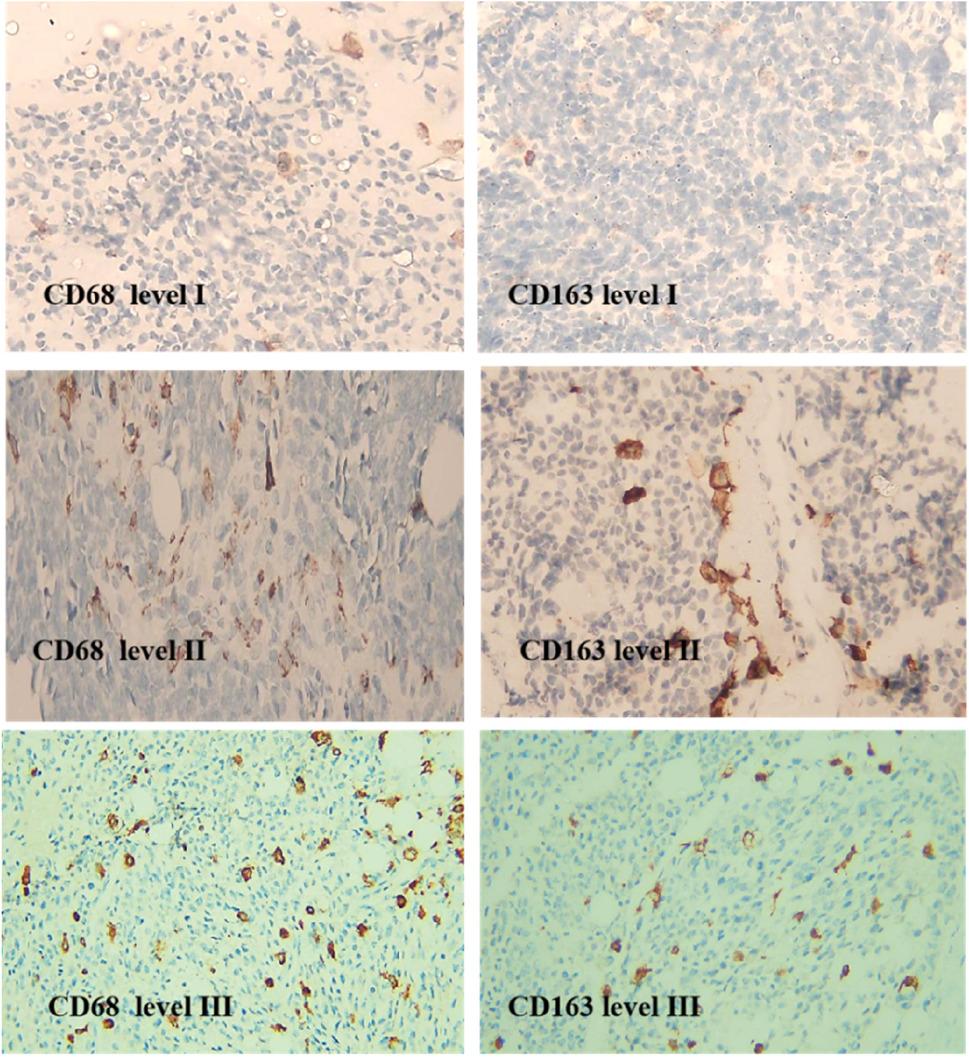
Immunohistochemical slides showing levels of expression of CD68 & CD163 (magnification power x200). Scoring of CD68 staining in tumor cells was performed as follows: I. <60 positive cells II. 61-130 positive cells, III. >130 positive cells. Scoring of CD163 staining in tumor cells was performed as follows: I. <80 positive cells, II. 81-140 positive cells, III. > 140 positive cells

The expression of CD68 and CD163 was evaluated by immunohistochemistry and analyzed using both quantitative and semi-quantitative approaches. The first method assessed the number of positive cells per 1 mm². For CD68, the scoring was based on the classification by Handl et al. (2018) [4] as follows: score I (< 60 positive cells) was identified in 41 cases (55.4%); score II (61–130 positive cells) in 21 cases (28.4%); and score III (> 131 positive cells) in 12 cases (16.2%). Similarly, CD163 expression per 1 mm² was categorized as follows: score I (< 80 positive cells) was observed in 30 cases (40.5%), score II (81–140 cells) in 17 cases (21.0%), and score III (> 140 cells) in 27 cases (36.5%) (Fig. 2).

The second method is by using the median for CD68; the median for CD68 is 60, so cases are classified into 2 groups: ≤60 positive cells include 41 cases (55.4%); >60 positive cells include 33 cases (44.6%). The median for CD163-positive cells is 90, so cases are classified into 2 groups: ≤90 positive cells include 38 cases (51.4%), and > 90 positive cells include 36 cases (48.6%) (Table 2).

**Table 2:** Summary of CD68 and CD163 expression methods

Characteristics (*n*, 74)	Number (%)	
CD68 level		
CD68 median 60, (Min-Max) (15–285)		
≤ 60		41 (55.4)
> 60		33 (44.6)
CD68 score		
I. ≤ 60		41 (55.4)
II. > 60 - ≤130		21 (28.4)
III. > 130		12 (16.2)
CD163 median 90, (Min-Max) (5-350)		
CD163 level		
≤ 90		3(51.4)8
> 90		36 (48.6)
CD163 score		
I. ≤ 80		30 (40.5)
II. > 80 - ≤140		17 (23)
III. > 140		27 (36.5)

Data presented as numbers and percentages as appropriate

### Chemotherapy given and Post induction chemotherapy evaluation 

All patients received systemic chemotherapy according to the COG study (COG-AEWS0031, CDV alternating with IE). Except for 4 patients, one patient escaped after confirmation of diagnosis, while 3 patients died post first cycle. The majority of the patients (50/70, 71.4%) received interval compression chemotherapy (every 2 weeks), while the rest (20, 28.6%) received non-interval compression chemotherapy (every 3 weeks). Objective response (CR/PR) was documented in the majority of patients (42/59, 71.2%), while non-objective response (SD/PD) was reported in 28.8% of patients (17/59). Fifteen patients were excluded from treatment response assessment (2 patients escaped, 6 patients died before the evaluation time, and 7 patients had non-conclusive measurements).

### Local control modalities

Local control was attempted in 86.5% (64/74); 37.5% (24 patients) underwent surgical resection, 37.5% (24 patients) received radiotherapy, and 11.5% (16 patients) had both modalities. Delay in local control was noticed in 46/64 (71.9%). Ten patients (13.5%) did not undergo local control treatment (5 died before local control, 2 escaped, 2 had PD, and 1 refused local control).

Of the patients, 54.1% (40/74) attempted surgical local control; 3/40 underwent upfront surgical excision, and out of 37 patients, 51.4% (19/37) had tumor necrosis < 90%, while 48.6% (18/37) had tumor necrosis > 90%; 1/40 had an inconclusive comment on surgical margin, and out of 39 patients, 84.6% (33/39) had negative surgical margin, while 15.4% (6/39) had positive surgical margin.

Regarding radiotherapy, of the 40 patients who had local radiation therapy (either by itself or in conjunction with surgery), 29 (72.5%) received ≥ 45 Gy and 11 (27.5%) received < 45 Gy. Lung radiation was given to 69.2% (9/13) of the thirteen patients who had lung metastases.

### Deaths

After a median follow-up duration of 34.4 months (range, 0.3–107 months), 26 out of 74 patients (35.1%) experienced either disease progression or recurrence. Among these, 18 patients (69.2%) developed disease progression, while 8 patients (30.8%) had a recurrence. The majority of these events (22/26, 84.6%) occurred within the first two years following the initial diagnosis.

A total of 34 deaths were recorded. Among the deceased, 19 patients (55.8%) were female, 31 (91.2%) had tumors of bony origin, and only 3 (8.8%) had extraskeletal disease. Eighteen patients (52.9%) presented with metastatic disease at diagnosis. Twelve patients (35.3%) had received non-interval compressed chemotherapy. CD68 expression ≤ 60 positive cells/mm² was noted in 19 patients (55.8%), and CD163 expression ≤ 90 positive cells/mm² was observed in 17 patients (50%).

Of the 34 deaths, 22 (64.7%) were attributed to disease-related mortality, while 12 (35.3%) were due to treatment-related toxicities, primarily infections. Among the 40 surviving patients, 35 (87.5%) were alive with no evidence of disease, whereas 5 (12.5%) remained alive with persistent disease.

### CD68 expression in patients with ES in relation to different prognostic factors

The younger age (< 15 years old) is associated with a higher median number for CD68 than the older group (≥ 15 years) (CD68 median, 65 vs. 55, respectively, *p* = 0.035) (Table 3).

**Table 3:** CD68 median level expression and scoring in patients with ES in relation to different prognostic factors

Characteristics	*N*	CD68			CD68 level (%) ^#^			CD68 scoring (%)			
		Median	Min-Max	*p*-value	≤ 60	> 60	*p*-value	≤ 60	> 60 - ≤130	> 130	*p*-value
Whole group	74	60.0	15–285		---	---	---	---	---	---	---
Gender											
Male	32	60.0	20–250	0.4	17 (53.11)	15 (46.9)	0.73	17 (53.1)	8 (25)	7 (21.9)	0.49
Female	42	60.0	15–285		24 (57.1)	18 (42.9)		24 (57.1)	13 (31)	5 (11.9)	
Age											
< 15 years	47	65.0	25–285	0.035	23 (48.9)	24 (51.1)	0.14	23 (48.9)	16 (34)	8 (17)	0.28
≥ 15 years	27	55.0	15–220		18 (66.7)	9 (33.3)		18 (66.7)	5 (18.5)	4 (14.8)	
Primary tumor site											
Axial bone	15	80	20–250	0.052	7 (46.7)	8 (53.3)	0.068	7 (46.7)	3 (20)	5 (33.3)	0.061
Extremity bone	44	57.5	15–285		29 (65.9)	15 (34.1)		29 (65.9)	11 (25)	4 (9.1)	
Soft tissue	15	50	25–230		5 (33.3)	10 (66.7)		5 (33.3)	7 (46.7)	3 (20)	
Tumor size											
< 8 cm	35	70	20–250	0.1	14 (40)	21 (60)	0.031	14 (40)	13 (37.1)	8 (22.9)	0.095
≥ 8 cm	35	55	15–285		23 (65.7)	12 (34.3)		23 (65.7)	8 (22.9)	4 (11.4)	
Metastatic status											
Non-metastatic	50	60	15–285	0.7	26 (52)	24 (48)	0.395	26 (52)	17 (34)	7 (14)	0.28
Metastatic	24	60	25–250		15 (62.5)	9 (37.5)		15 (62.5)	4 (16.7)	5 (20.8)	
CD163 level											
≤ 90	38	---	---	---	27 (71.1)	11 (28.9)	0.005	27 (71.7)	6 (15.8)	5 (13.2)	0.016
> 90	36	---	---		14 (38.9)	22 (61.1)		14 (38.9)	15 (41.7)	7 (19.4)	
CD163 score											
≤ 80	30	---	---	---	---	---	---	19 (63.3)	6 (20)	5 (16.7)	0.001
> 80-≤140	17	---	---		---	---		15 (88.2)	1 (5.9)	1 (5.9)	
> 140	27	---	---		---	---		7 (25.9)	14 (51.9)	6 (22.2)	
Dose intensity											
Interval compression	50	60	20–285	0.92	26 (52)	24 (48)	0.323	26 (52)	14 (28)	10 (20)	0.518
Non-interval compression	20	60	15–250		13 (65)	7 (35)		13 (65)	5 (25)	2 (10)	
Disease status post induction											
CR	13	100	25–250	0.054	5 (38.5)	8 (61.5)	0.393	5 (38.5)	4 (30.8)	4 (30.8)	0.277
PRI < 70% (17)	29	60	20–250		8 (47.1)	9 (52.9)		8 (47.1)	7 (41.2)	2 (11.8)	
PRII ≥ 70% (12)					8 (66.7)	4 (33.3)		8 (66.7)	1 (8.3)	3 (25)	
SD	9	60	15–115		6 (66.7)	3 (33.3)		6 (66.7)	3 (33.3)	0	
PD	8	55	30–75		6 (75)	2 (25)		6 (75)	2 (25)	0	
Type of local control											
Radiotherapy	24	60	25–250	0.3	13 (54.1)	11 (45.8)	0.856	13 (54.2)	6 (25)	5 (20.8)	0.306
Surgery	24	60	35–140		14 (58.3)	10 (41.7)		14 (58.3)	9 (37.5)	1 (4.2)	
Combined	16	60	15–240		9 (56.3)	7 (43.7)		9 (56.2)	3 (18.8)	4 (25)	
Tumor necrosis (post induction chemotherapy)											
< 90%	19	60	15–240	0.7	12 (63.2)	7 (36.8)	0.898	12 (63.2)	5 (26.3)	7 (10.5)	1
≥ 90%	18	57.5	30–220		11 (61.1)	7 (38.9)		11 (61.1)	6 (33.3)	1 (18)	

*CR* complete response, *PRI* partial response (volume reduction <70-50%), *PRII* partial response (volume reduction <100-≥70%), *SD* stable disease, *PD* progressive disease

^#^Absolute (relative frequency)/median (minimum-maximum), data presented as numbers and percentages as appropriate

CD68 level was affected by initial tumor size and CD163 level. Smaller tumor size (< 8 cm) is associated with elevated CD68 level (> 60), and vice versa (*p* = 0.03). Also, elevated CD68 (> 60) is associated with elevated CD163 levels (> 90), and vice versa (*p* = 0.005). There was a significant positive fair correlation between CD68 and CD163 (*p* = 0.002, *r* = 0.362). Also, high CD68 scoring is associated with high CD163 scoring, and vice versa (*p* = 0.001) (Table 3).

The median number of CD68 showed a near-significant relation with primary tumor site (*p* = 0.052) and type of response post-induction treatment (*p* = 0.054). Axial bone location is associated with a higher median number for CD68 than extremity bone or soft tissue sites (CD68 median, 80, 57.5, and 50, respectively). Complete response to induction chemotherapy is associated with a high median number for CD68 (100) as compared with those with poor response types (60 in PR, 60 in SD, and 55 in PD, respectively) (Table 3).

There was no significant correlation with other clinicopathologic variables, including gender, metastatic status, chemotherapy dose intensity, type of local control needed, and tumor necrosis % (Table 3).

### CD163 expression in patients with ES in relation to different prognostic factors

On the other side, the CD163 level was affected by the patients’ age and CD68 level. Younger age (< 15 years) is associated with elevated CD163 level (> 90), and vice versa (*p* = 0.04). Also, elevated CD163 (> 90) is associated with elevated CD68 levels (> 60), and vice versa (*p* = 0.005). Also, high CD163 scoring is associated with high CD68 scoring, and vice versa (*p* = 0.001) (Table 4).

**Table 4:** CD163 median level expression and scoring in patients with ES in relation to different prognostic factors

Characteristics	*N*	CD163			CD163 level (%) ^#^			CD163 scoring (%)			
		Median	Min-Max	*p*-value	≤ 90	> 90	*p*-value	≤ 80	> 80 - ≤140	> 140	*p*-value
Whole group	74	90.0	5–350	---	---	---	---	---	---	---	---
Gender											
Male	32	97.5	10–300	0.2	15 (46.9)	17 (53.1)	0.501	11 (34.4)	6 (18.8)	15 (46.9)	0.265
Female	42	90.0	5–350		23 (54.8)	19 (45.2)		19 (45.2)	11 (26.2)	12 (28.6)	
Age											
< 15 years	47	110.0	5–350	0.2	20 (42.6)	27 (57.4)	0.046	17 (36.2)	11 (23.4)	19 (40.4)	0.558
≥ 15 years	27	90.0	10–260		18 (66.7)	9 (33.3)		13 (48.1)	6 (22.2)	8 (29.6)	
Primary tumor site											
Axial bone	15	100	10–300	0.6	7 (46.7)	8 (53.3)	0.801	5 (33.3)	5 (33.3)	5 (33.3)	0.871
Extremity bone	44	90	5-350		24 (54.5)	20 (45.5)		19 (43.2)	9 (20.4)	16 (36.4)	
Soft tissue	15	50	10–250		7	8		6 (40)	3 (20)	6 (40)	
Tumor size											
< 8 cm	35	140	5-300	0.1	16 (45.7)	19 (54.3)	0.231	13 (37.1)	7 (20)	15 (42.9)	0.449
≥ 8 cm	35	90	10–350		21 (60)	14 (40)		17 (48.6)	8 (22.9)	10 (28.6)	
Metastatic status											
Non-metastatic	50	90	5-350	0.6	26 (52)	24 (48)	0.872	21 (42)	10 (20)	19 (38)	0.680
Metastatic	24	92	10–260		12 (50)	12 (50)		9 (37.5)	7 (29.2)	8 (33.3)	
CD68 level											
≤ 60	41	---	---	---	27 (65.9)	14 (34.1)	0.005	19 (46.3)	15 (36.6)	7 (17.1)	< 0.001
> 60	33	---	---		11 (33.3)	22 (66.7)		11 (33.3)	2 (6.1)	20 (60.6)	
CD68 score											
≤ 60	41	---	---	---	---	---	---	19 (46.3)	15 (36.6)	7 (17.1)	0.001
> 60-≤130	21	---	---		---	---		6 (28.6)	1 (4.8)	14 (66.7)	
> 130	12	---	---		---	---		5 (41.7)	1 (8.3)	6 (50)	
Dose intensity											
Interval compression	50	87.5	10–350	0.2	27 (54)	23 (46)	0.496	24 (48)	8 (16)	18 (36)	0.064
Non-interval compression	20	97.5	5-260		9 (45)	11 (55)		4 (20)	7 (35)	9 (45)	
Disease status post induction											
CR	12	150	10–280	0.1	5 (38.5)	8 (61.5)	0.609	4 (30.8)	2 (15.4)	7 (53.8)	0.603
PRI < 70% (17)	29	90	10–260		9 (52.9)	8 (47.1)		7 (41.2)	2 (11.8)	8 (47.1)	
PRII ≥ 70% (12)					6 (50)	6 (50)		5 (41.7)	4 (33.3)	3 (25)	
SD	9	140	10–210		4 (44.4)	5 (55.6)		3 (33.3)	2 (22.2)	4 (44.4)	
PD	8	85	10–190		6 (75)	2 (25)		4 (50)	3 (37.5)	1 (12.5)	
Type of local control											
Radiotherapy	24	90	10–280	0.7	13 (54.2)	11 (45.8)	1	9 (37.5)	7 (29.1)	8 (33.3)	0.787
Surgery	24	85	5-260		13 (54.2)	11 (45.8)		12 (50)	4 (16.7)	8 (33.3)	
Combined	16	90	10–250		9 (56.2)	7 (43.8)		5 (31.2)	4 (25)	6 (37.5)	
Tumor necrosis (post induction chemotherapy)											
< 90%	19	90	10–260	0.2	10 (52.6)	9 (47.4)	0.858	8 (42.1)	3 (15.8)	8 (42.1)	0.582
≥ 90%	18	90	5-170		10 (55.6)	8 (44.4)		8 (44.4)	5 (27.8)	5 (27.8)	

*CR* complete response, *PRI* partial response (volume reduction <70-50%), *PRII* partial response (volume reduction <100-≥70%), *SD* stable disease, *PD* progressive disease

^#^Absolute (relative frequency)/median (minimum-maximum), data presented as numbers and percentages as appropriate

There was no significant correlation with other clinicopathologic variables, including gender, primary tumor site, tumor size, metastatic status, chemotherapy dose intensity, type of response post-induction treatment, type of local control needed, and tumor necrosis % (Table 4).

### Survival analysis

**Fig. 3:**
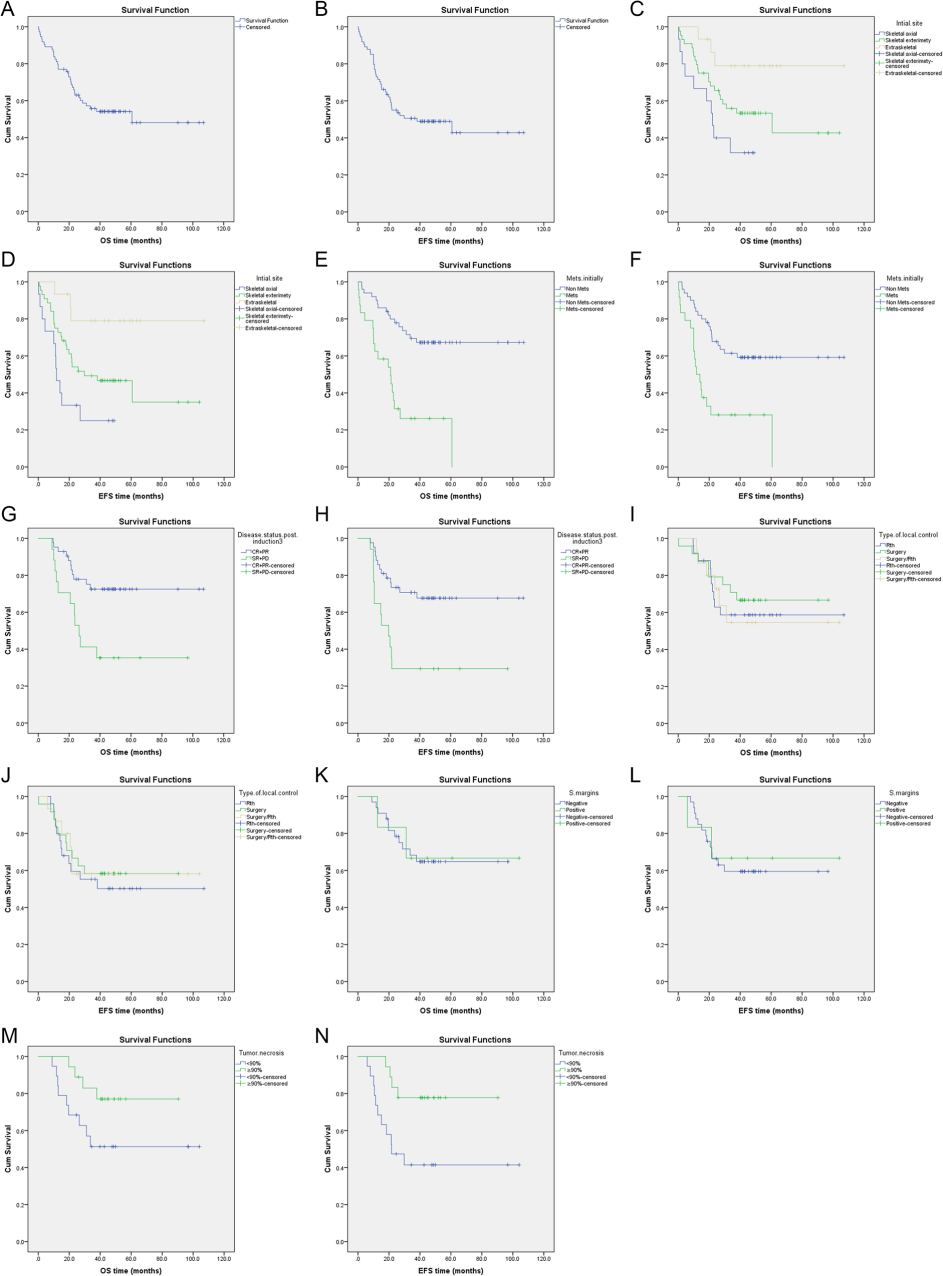
Kaplan–Meier estimates of OS (**A**) and EFS (**B**) in relation to different prognostic factors (**C** - **N**) among patients with ES

For the entire study group, 34 patients died, the median overall survival was 60 months, and the 3-year OS and EFS estimates were 55.7% and 50.6%, while the 5-year OS and EFS estimates were 48% and 42.8% (Fig. 3A & B).

There was significant correlation between outcome in terms of OS and EFS and initial tumor site (skeletal vs. extra skeletal tumors), *p* = 0.022 & *p* = 0.004, respectively (Figs. 3 C & D); tumor stage (localized vs. metastatic), *p* = < 0.001 & *p* = < 0.001, respectively (Figs. 3E & F); and disease status post induction treatment (CR/PR vs. SD/PD), *p* = 0.006 & *p* = 0.002, respectively (Figs. 3G & H).

Also, there was no significant correlation between outcome in terms of OS and EFS and local control type (surgical resection vs. radiation therapy vs. combined), *p* = 0.768 & *p* = 0.850, respectively (Fig. 3I & J), and surgical margin (positive vs. negative), *p* = 0.957 & *p* = 0.819, respectively (Fig. 3K & L).

Histological response (tumor necrosis %; ≥90% vs. < 90%) in the tumor specimen post-induction chemotherapy showed no statistically significant impact in terms of OS, *p* = 0.074 (Fig. 3M), while there was a statistically significant difference in terms of EFS, *p* = 0.015 (Fig. 3N).

In the multivariate Cox regression analysis, the initial tumor stage and post-induction chemotherapy disease status were independent factors significantly influencing OS and EFS.

Patients with metastatic disease had poorer OS (HR: 5.7, 95% CI: 2.270–14.667, *P* < 0.001) & EFS (HR: 4.085, 95% CI: 1.633–10.219, *P* = 0.003) compared to those with localized disease. Also, OS and EFS were adversely affected by the poor response (SD/PD) to induction chemotherapy OS (HR: 3.087, 95% CI: 1.288–7.396, P 0.011) & EFS (HR: 4.869, 95% CI: 1.958–12.108, *P* = 0.001) compared to the good response (CR/PR).

#### OS & EFS was studied in relation to levels of CD 68 & CD 163

**Fig. 4:**
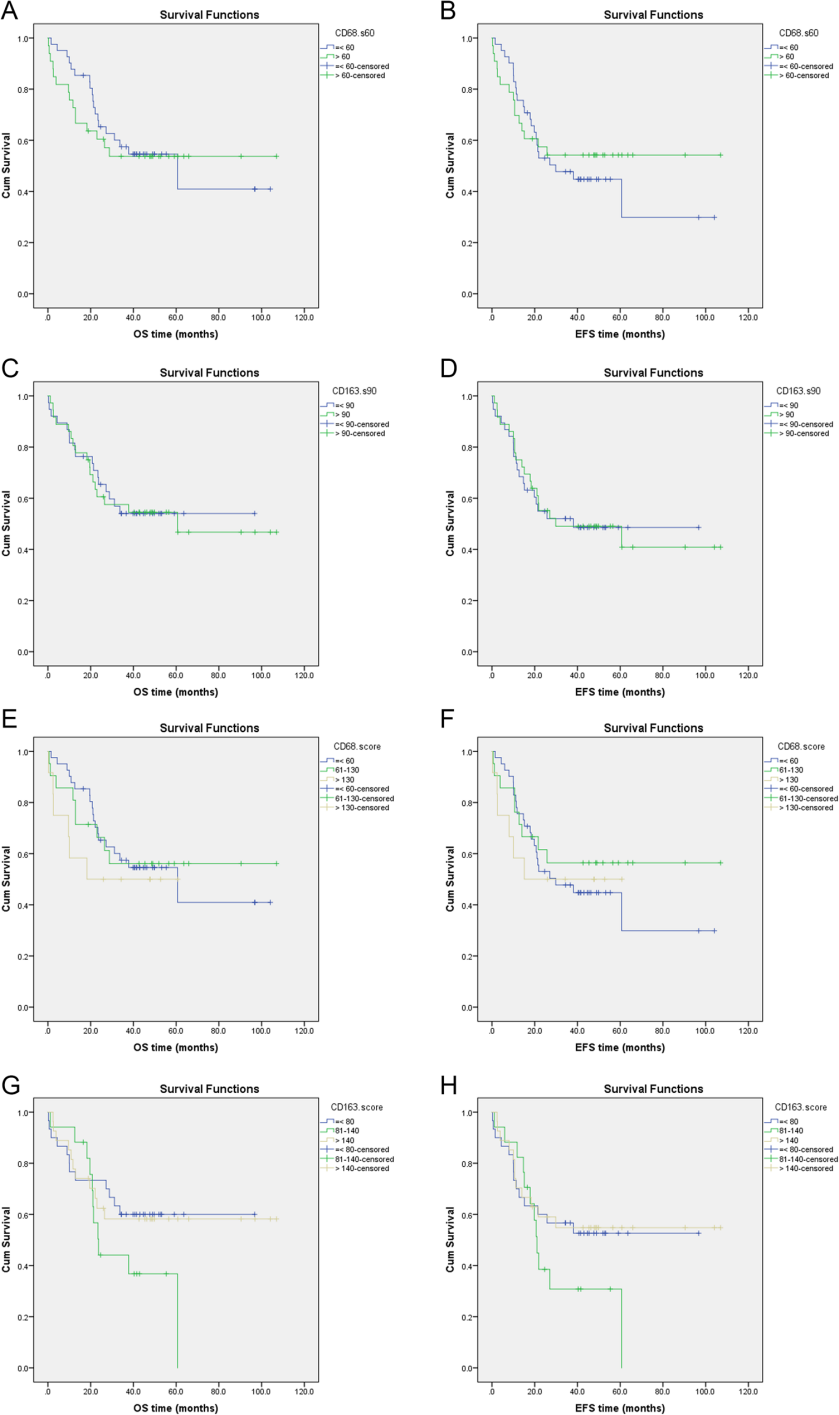
Kaplan-Meier survival curves for OS and EFS in relation to CD68 & CD163 median levels (**A**-**D**); CD68 & CD163 scoring levels (**E**-**H**), respectively, among patients with ES

There was no significant correlation between outcome in terms of OS and EFS and CD68 median level (≤60 vs. >60), *p* = 0.744 & p = 0.636, respectively (Fig. 4A & B), either scoring levels (≤60 vs. >60-≤130 vs. > 130), *p* = 0.693 & p = 0.707, respectively (Fig. 4E & F). 

Also, there was no significant correlation between outcome in terms of OS and EFS and CD163 median level (≤ 90 vs. > 90), *p* = 0.912 & p = 0.950, respectively(Fig. 4C & D), either scoring levels (≤80 vs. >80-≤140 vs. > 140), *p* = 0.293 & p = 0.346, respectively (Fig. 4G & H).

In the multivariate Cox regression analysis, both the CD68/163 median level and score were not statistically significantly associated with OS and EFS.

## Discussion

TAMs exhibit considerable heterogeneity, making their role within the tumor microenvironment highly complex. TAMs exist along a spectrum of polarization states, ranging from the classically activated M1 phenotype, which exerts anti-tumor effects, to the alternatively activated M2 phenotype, which promotes tumor progression. The relative abundance and spatial distribution of these phenotypes within the tumor tissue influence the net impact of TAMs on tumor biology. Consequently, their effect on patient outcomes may be either beneficial or detrimental [4].

Over the years, numerous studies have explored the prognostic significance of TAMs in solid tumors. Most have reported that high TAM infiltration supports tumor growth and correlates with unfavorable prognosis [14]. However, the literature includes both supportive and contradictory findings, highlighting the need for quantitative analyses to clarify these associations. In a comprehensive meta-analysis, Zhang et al. demonstrated that a high density of TAMs, assessed by immunohistochemistry, was significantly associated with poorer overall survival in solid tumors, with a pooled relative risk (RR) of 1.15 [14].

TAMs contribute significantly to tumor progression through the secretion of growth factors, cytokines, chemokines, and proteolytic enzymes. These mediators facilitate key processes such as tumor invasion, angiogenesis, and metastasis. In ES, the presence of infiltrating TAMs has been linked to systemic inflammation, increased tumor vascularization, and poorer clinical outcomes, suggesting their potential utility as a prognostic marker in this tumor type [15].

In this retrospective analysis of 74 patients diagnosed with ES, we assessed the prognostic significance of TAMs in relation to survival outcomes and various clinical and pathological parameters. The demographic and clinical features of our cohort were largely in line with those reported in previous studies evaluating the role of TAMs in ES. Specifically, our results are consistent with the findings of Handl et al. and Fujiwara et al., as all three studies demonstrated a similar median age at diagnosis, comparable male-to-female ratios, and a substantial proportion of patients presenting with metastatic disease [4, 15]. This consistency across studies supports the representativeness of our cohort.

In our study, CD68 expression showed a significant association with both patient age and tumor size at diagnosis. Higher CD68 levels were more frequently observed in patients younger than 15 years (*p* = 0.035), and elevated CD68 expression (> 60) was significantly associated with smaller tumor size (< 8 cm) (*p* = 0.03). These findings differ from those reported by Fujiwara et al. and Handl et al., who did not identify any significant correlation between CD68 levels and either age or tumor size [4, 15].

We also observed that patients with axial skeletal tumors had higher median CD68 levels compared to those with tumors in the extremities or extraskeletal sites (median CD68: 80, 57.5, and 50, respectively), with a borderline statistical significance (*p* = 0.052). Additionally, higher CD68 expression (median: 100) appeared to be associated with a favorable response to induction chemotherapy (CR), whereas lower CD68 levels (≤ 60) were more frequently seen in patients with less favorable outcomes (PR, SD, or PD). This association also approached statistical significance (*p* = 0.054). Again, these trends were not observed in previous studies by Fujiwara et al. and Handl et al. [4, 15]. This could suggest a role for macrophages in the early inflammatory response to chemotherapy in patients with ES, which needs further testing in larger studies.

Other clinical factors, including gender, metastatic status at diagnosis, chemotherapy dose intensity, type of local control (surgery, radiotherapy, or combined), and post-chemotherapy tumor necrosis (< 90% vs. ≥90%), did not show a statistically significant association with CD68 expression in our analysis.

Our study revealed that CD163 expression was significantly influenced by patient age at diagnosis. Higher CD163 levels (> 90) were more commonly observed in patients younger than 15 years (*p* = 0.04). This finding contrasts with the results of Handl et al., who reported no association between CD163 expression and age [4].

Other clinical parameters—including gender, tumor size, metastatic status, dose intensity, response after induction therapy, type of local control, and post-chemotherapy tumor necrosis—did not show significant differences in relation to CD163 expression levels in our cohort. However, in contrast to our findings, Handl et al. noted a significant association between higher CD163 + TAM infiltration and localized disease.

A notable observation in our study was the significant correlation between CD68 and CD163 expression. Higher CD68 levels (> 60) were significantly associated with higher CD163 levels (> 90), and vice versa. Specifically, 61.1% of patients with CD163 > 90 had CD68 > 60, compared to 28.9% of those with CD163 ≤ 90 (*p* = 0.005). Similarly, 66.7% of patients with CD68 > 60 had CD163 > 90, compared to 34.1% with CD68 ≤ 60 (*p* = 0.005). This mutual association was further supported by a fairly positive correlation (*r* = 0.362, *p* = 0.002), suggesting a potential co-regulation or shared biological role of these two macrophage markers in the tumor microenvironment.

This positive fair correlation between CD68 and CD163, reflects the fact that both markers identify overlapping macrophage populations in ES. CD163 is not exclusively restricted to M2-polarized macrophages, and macrophages within ES frequently exhibit mixed or partial polarization states, leading to co-expression of CD68 and CD163.

Despite this correlation in macrophage density, neither marker demonstrated prognostic significance. This likely reflects the limited functional specificity of CD68 and CD163 in ES, where macrophage phenotype does not follow the classical M1/M2 paradigm and where reactive inflammatory infiltration may not mirror tumor aggressiveness. This lack of prognostic significance may be partly attributable to the limited sample size and the low number of events, which reduce the statistical power of survival analyses. In small cohorts, hazard ratios become unstable, and subtle differences may fail to reach significance, leading to a potential type II error.

Additionally, CD68 expression showed a borderline significant association with primary tumor site (*p* = 0.052) and response to induction chemotherapy (*p* = 0.054), which may warrant further investigation.

In our study, no significant association was found between EFS or OS and the levels of CD68 (≤ 60 vs. >60) or CD163 (≤ 90 vs. >90) expression. These findings are consistent with those reported by Handl et al., who also found no statistically significant relationship between CD68-positive macrophages and disease stage or survival outcomes in ES [4]. However, their analysis did reveal that higher levels of CD163-positive TAMs were associated with localized disease and a trend toward improved survival.

In contrast, Lau et al. demonstrated that TAMs in ES of bone can differentiate into osteoclasts through both Receptor activator of NF-kB ligand R(ANKL)-dependent and RANKL-independent pathways. Their findings suggest that TAMs may contribute to tumor progression and osteolysis by enhancing osteoclastogenesis [16].

Fujiwara et al. further supported the prognostic significance of TAMs in ES through multivariate analysis, identifying a strong correlation between TAM infiltration and poor clinical outcomes. They proposed that TAMs promote disease progression by stimulating angiogenesis and osteoclastogenesis via cytokine and chemokine release, highlighting their potential as therapeutic targets [15]. In a separate analysis, Fujiwara et al. also found that high TAM infiltration, increased microvascular density, elevated white blood cell counts (> 6,800/µL), and higher C-reactive protein levels (> 0.2 mg/dL) were significantly associated with worse prognosis [10].

Stahl et al. expanded on this by identifying immune-related prognostic markers in ES. Their multivariate Cox regression analysis revealed that activated natural killer cells and the combined presence of M2 macrophages, neutrophils, and T cells were the most significant independent predictors of EFS and OS. Their findings confirmed that macrophages represent a major immune cell subset in ES family tumors, consistent with immunohistochemical analyses in pediatric cancers [17].

Consistent with Handl et al., our study also found that CD163-positive TAMs were more abundant than CD68-positive cells (median 90 vs. 60, respectively). Similarly, Handl et al. reported higher median levels of CD163 (130) compared to CD68 (96). In our cohort, 48.6% and 59.5% of cases showed moderate to high infiltration of CD68 + and CD163 + macrophages, respectively, which is comparable to the 71% and 79% reported by Handl et al. [4]. These similarities further support the relevance of TAMs in the tumor microenvironment of ES, though their prognostic impact remains variable across studies.

The spatial distribution (intratumoral vs. peripheral) and the activation/polarization status of TAMs may be biologically more relevant than total macrophage density alone. In our cohort, neither CD68+ (pan macrophage marker) nor CD163+ (M2-like/alternatively activated macrophage) macrophage density was significantly associated with survival, in contrast to Fujiwara et al., who reported that higher CD68 + infiltration predicted poorer prognosis in ES [15]. This discrepancy may reflect methodological differences, including patient population, sample size, and the limited assessment of macrophage functional phenotype and spatial distribution. These findings suggest that macrophage spatial distribution and activation status, rather than total density alone, may be more critical determinants of clinical impact, consistent with transcriptomic studies such as Stahl et al., highlighting the prognostic relevance of M2-like macrophages and overall immune composition [17].

Fujiwara et al., demonstrated that increased CD68 + macrophage infiltration was associated with poor prognosis in ES [15]. In contrast, our study evaluated both CD68 and CD163 expression, allowing partial assessment of macrophage polarization toward an M2-like phenotype. This distinction may contribute to differences in prognostic associations observed between studies and supports the concept that macrophage activation status may be more informative than total macrophage density alone.

In addition, macrophage polarization status (e.g., pro-inflammatory M1-like vs. immunosuppressive M2-like phenotypes) may critically determine their prognostic relevance. Studies such as Fujiwara et al., reported different associations between macrophage infiltration and clinical outcome, potentially reflecting methodological differences in marker selection and phenotype characterization [15]. Variability in antibodies (e.g., CD68 vs. CD163), scoring systems, patient populations, and treatment regimens may further contribute to the discrepancies observed across studies.

Another study performed by stahl et al., used gene expression deconvolution (CIBERSORT) to profile immune infiltrates in a large dataset of ESFTs. They identified distinct macrophage subsets, including M1- and M2-like populations, and showed that higher M2-like macrophage abundance was associated with poorer prognosis. Their study highlighted that macrophage phenotype and overall immune composition, rather than total macrophage density alone, may be critical determinants of clinical outcome [17].

In our study, the 3 OS and EFS rates for patients with ES were 55.7% and 50.6%, respectively. These outcomes are comparable to those reported in several previous studies. For example, Çakmakcı et al. observed a 3-year OS of 54% and EFS of 34%, while Majeed et al. reported a 3-year OS of 45% and EFS of 25%. Similarly, Mahmoud et al. found OS and EFS rates of 47.5% and 36%, respectively, and Pant et al. reported even lower 4-year OS and EFS rates of 23.5% and 21.5% [18–21].

In contrast, other studies have demonstrated more favorable survival outcomes. Gupta et al. reported a 3-year OS of 81% ± 7.7%, Hsu et al. found a 3-year OS of 67.4%, Wan et al. reported a 5-year OS of 62.4%, and Jakutis et al. observed a 5-year OS and EFS of 65% and 61%, respectively [22–25].

Studies of CD68⁺ and CD163⁺ TAMs in other tumors show variable roles depending on tumor type. In many adult carcinomas, including head and neck, breast, renal, and oral squamous cell carcinomas, high CD163⁺ TAM density is generally associated with tumor progression, metastasis, and poor survival, reflecting a cancer-promoting, M2-like phenotype. CD68⁺ TAMs are often less prognostic but may contribute to tumor growth in certain contexts [26–29]. In pediatric tumors, findings are more heterogeneous: in neuroblastoma, hepatoblastoma, and pediatric Hodgkin lymphoma, high CD163⁺ TAMs correlate with aggressive disease and worse outcomes, while in osteosarcoma and medulloblastoma, higher TAM density has been linked to better outcomes, suggesting potential anti-tumor activity [30–32]. Overall, TAMs can exert either cancer-promoting or potentially protective effects depending on tumor type, microenvironment, and patient context, which is consistent with our observation in ES, where increased TAM density did not significantly impact survival.

The variability in survival outcomes across studies may be attributed to differences in patient populations, treatment protocols, and access to advanced supportive care. Additionally, the complex role of TAMs in the tumor microenvironment may contribute to this variability. The phenotypic diversity of TAMs likely plays a key role in modulating tumor behavior. While several studies support their tumor-promoting functions, others have demonstrated potential tumor-suppressive effects [4].

A substantial proportion of deaths in our cohort were related to treatment rather than disease progression. This treatment-related mortality can act as a competing risk, potentially masking the true prognostic impact of tumor-associated macrophages (CD68 and CD163); patients with high macrophage infiltration who died from treatment complications may decrease the apparent association between macrophages and survival outcomes. Given the substantial treatment-related mortality, the lack of statistically significant associations between TAM infiltration and survival may in part reflect the confounding effect of competing risks rather than a true absence of biological effect.

Therefore, the lack of a clear correlation in our study may partly reflect this competing risk. Future studies using larger cohorts and statistical methods that account for treatment-related mortality could better clarify the biological significance of macrophage infiltration in pediatric Ewing sarcoma. While our primary analyses focused on overall survival, future studies could consider competing risk models (e.g., Fine-Gray subdistribution hazard models) to specifically account for treatment-related mortality and better isolate the prognostic impact of tumor-associated macrophages.

As a key finding, our analysis revealed multiple important factors that affect survival outcome: initial primary tumor site, tumor stage, post-induction chemotherapy disease status, and histological response to induction chemotherapy.

Regarding the initial primary tumor site, patients with extraskeletal tumors had better survival outcomes (OS, 79% & EFS, 79%) than those with extremity or axial tumors (OS, 55.9% & 32% & EFS, 49.3% & 25%, respectively) with statistical significance (*p* = 0.022 & 0.004, respectively); this is similar to different studies that show patients with extraskeletal ES have more favorable outcomes compared with the skeletal subtype [33–35].

As for tumor stage, metastatic status at time of diagnosis had a statistically significant effect on OS & EFS, *p* = < 0.001 & *p* = < 0.001, respectively. Patients with localized disease have better outcomes in comparison to patients with metastatic disease. The data gathered in the studies strongly concluded that primary metastatic ES has a poor prognosis [36–38].

As for the response type to induction chemotherapy, patients with an early good response (CR/PR) have a better survival outcome than those with a poor response (SD/PD), with a statistically significant difference in terms of OS and EFS, *p* = 0.006 & *p* = 0.002, respectively.

Our data was in line with what was reported in a COG study on a large group of treated patients with ES; the choice of the mode of local control was not related significantly to EFS, overall survival, or distant failure, although the risk of local failure was greater for radiation compared with surgery. These data support surgical resection when appropriate, whereas radiotherapy remains a reasonable alternative in selected patients [39].

In contrast to different studies, our study did not show a significant correlation between survival and surgical margins, although Fritz et al. and Bacci et al. reported better 5-year EFS and OS rates of patients with negative surgical margins as compared to those with positive margins [36, 40].

Regarding the impact of histological response, our findings showed that tumor necrosis ≥ 90% in the tumor specimen post induction chemotherapy significantly improves EFS (*p* = 0.015); however, OS is not associated with the same significance (*p* = 0.074). Similarly, Bosma et al. showed that histological response (necrosis of at least 90%) had high prognostic value in terms of EFS, but evidence for a clear association with OS is less consistent [41].

The multivariate Cox regression analysis demonstrated that initial tumor stage and post-induction chemotherapy disease status were independent factors significantly influencing OS and EFS. In agree with what was published by Fritz et al. and Ladenstein et al. [36, 37].

The study has several limitations. The retrospective design. In addition, the limited amount of tissues in the study (core biopsies, especially in the existence of necrosis according to the histologic nature of the disease) hindered correlations of results according to spatial distribution. Future studies using spatial profiling are needed to clarify these aspects. Third limitation: our use of hotspot-based field selection may still cause variability because of tumor heterogeneity, even though we included multiple representative fields to reduce this effect. Fourth limitation: our primary analysis used median-based dichotomization for CD68 and CD163, while literature-based cutoffs were applied as exploratory comparisons. Although this dual approach allowed us to test robustness and align with prior studies, it also introduced the risk of multiple comparisons, which may affect reproducibility and generalizability of the findings. Finally, the absence of inter-observer variability analysis (discrepancies in measurement and interpretation).

In conclusion, TAM markers, CD68 and CD163-defined macrophage infiltration, demonstrated no significant prognostic impact on survival in terms of OS and EFS. Contrary to findings in some previous studies, our data revealed that higher TAM density was associated with younger patient age, smaller tumor size, skeletal primary tumor site, and complete response following induction chemotherapy.

## Data Availability

All data generated or analyzed during this study are included in this published article [and its supplementary information files].

## Abbreviations

ES: Ewing sarcoma
TAMs: Tumor-associated macrophages
NCI: National Cancer Institute
OS: Overall survival
EFS: Event-free survival
TME: Tumor micro-environment
CDV: Cyclophosphamide, doxorubicin, and vincristine
IE: Ifosfamide and etoposide
RECIST: Response evaluation criteria in solid tumors (RECIST criteria
HR: Hazard ratios
CI: Confidence intervals
RANKL: Receptor activator of NF-kB ligand
